# Acute hemorrhagic gastroenteritis caused by an “Asian” CPV-2c variant in a vaccinated dog from Slovakia (Central Europe): a case report

**DOI:** 10.3389/fvets.2026.1864309

**Published:** 2026-06-24

**Authors:** Patrícia Petroušková, Andrea Pelegrinová, Monika Drážovská, Anna Ondrejková, L'uboš Korytár, Marián Prokeš, Boris Vojtek, Jana Mojžišová Vaščinec, René Mandelík

**Affiliations:** Department of Epizootiology, Parasitology and Protection of One Health, University of Veterinary Medicine and Pharmacy in Košice, Košice, Slovakia

**Keywords:** Asian, canine parvovirus, Central Europe, CPV-2c, Slovakia, vaccination

## Abstract

An 8-month-old vaccinated male English Cocker Spaniel was presented with hemorrhagic diarrhea, vomiting, and lethargy. Canine parvovirus (CPV-2) infection was indicated by a weakly positive rapid antigen test and confirmed by a combination of serological, molecular, and virological methods. Serological analysis revealed a high IgM titer (1:2,700) and the absence of IgG antibodies, while hemagglutination inhibition (HI) and serum neutralization tests (SNT) confirmed the presence of functional antibodies (≥1:640). Molecular characterization based on the *VP2* gene (1,755 bp) identified the CPV-2c variant with amino acid signatures (5Gly, 267Tyr, 324Ile, 370Arg, and 440Thr) typical of the “Asian” lineage. The virus was successfully isolated in A-72 cell culture with characteristic cytopathic effects (CPE). Following intensive supportive therapy, the dog was discharged in stable condition. This report provides molecular evidence of the circulation of “Asian” CPV-2c variants in Central Europe and highlights the importance of comprehensive diagnostic approaches in vaccinated dogs.

## 1 Introduction

Canine parvovirus type 2 (CPV-2) remains one of the most important viral pathogens of dogs worldwide, causing acute gastroenteritis with high morbidity and mortality (>70% in puppies and < 1% in adult dogs) (1). CPV-2 had undergone continuous genetic evolution (0.0045 substitutions/site/year/*VP2* gene) (2), resulting in the global spread of three antigenic variants, including CPV-2a, CPV-2b, and CPV-2c. These variants differ primarily in the VP2 capsid protein (3), which plays a key role in host–receptor binding, antigenicity, and virus–host interactions (4, 5). In recent years, particular attention has been focused on the so-called “Asian” CPV-2c lineage, characterized by a specific amino acid signature in the VP2 protein (5G, 267Y, 324I, 370R, 440T). These amino acid residues have been previously reported in Asian strains (6–8) and subsequently in European (9–14) and African (15, 16) countries, supporting ongoing viral evolution and geographic expansion of this lineage in the global dog population (12).

Vaccination remains the cornerstone of CPV-2 prevention. Currently available attenuated vaccines are considered effective and capable of providing cross-protection against circulating CPV-2 variants (17–20). However, sporadic cases of CPV infection in vaccinated dogs continue to be reported (21–25), raising questions regarding the role of host-related factors, vaccination timing, and the potential impact of emerging viral variants on vaccine-induced immunity.

The present report describes a case of a vaccinated 8-month-old English Cocker Spaniel admitted for acute hemorrhagic gastroenteritis and confirmed for CPV-2 by a weakly positive antigen test result. Further serological, molecular, and virological investigations confirmed infection with the “Asian” CPV-2c variant. Following intensive supportive therapy, the dog was discharged after 7 days in stable condition. To the best of our knowledge, this is among the first reports providing molecular evidence of an “Asian” CPV-2c lineage associated with a clinical case in Central Europe and highlights its potential relevance in vaccinated dogs with clinical manifestations of CPV infection.

The objectives of reporting this case are to demonstrate (i) that parvovirus infection should remain a differential diagnosis in dogs presenting with clinical signs regardless of vaccination status; (ii) that weakly positive antigen test results require careful interpretation in context with clinical and laboratory findings; and (iii) the importance of molecular characterization of circulating CPV-2 variants in vaccinated clinical cases.

## 2 Case description

An 8-month-old male English Cocker Spaniel (date of birth: March 8, 2025) weighing 13.1 kg (Supplementary Figure 1A) was referred to the Clinical Workplace of Infectious and Parasitic Animal Diseases of the University of Veterinary Medicine and Pharmacy in Košice (November 17, 2025) for acute gastrointestinal disease characterized by lethargy, vomitus, and hemorrhagic diarrhea (Supplementary Figure 1B) lasting for more than 2 days. According to the owner, the dog was clinically healthy before the onset of symptoms and was the only pet in the household; it went for regular walks outdoors and had contact with other dogs.

The patient's vaccination history for CPV-2 was properly documented. The primary vaccination course was initiated at 7 weeks of age (April 29, 2025) with a DHPPi vaccine, followed by two vaccinations administered at 11 (May 26, 2025) and 15 weeks of age (June 23, 2025) using a DHPPi-Lmulti vaccine.

At admission, the dog was apathic but responsive. Physical examination revealed moderate dehydration with prolonged capillary refill time (>5 s) and abdominal discomfort on palpation. The patient's rectal temperature upon admission was 38.7 °C, and the dog remained afebrile throughout the entire course of hospitalization.

Initial suspicion of infection was guided by the patient's history and clinical presentation. Detailed results of the clinical examination are included in Supplementary Method 1. Clinical examination was performed according to the clinical examination protocol (26). Diagnosis was subsequently confirmed by clinical, molecular and serological investigations, supported by virus isolation in cell culture.

The dog was treated with intensive multimodal supportive therapy. Treatment included intravenous fluid therapy, antiemetic and gastroprotective medication, analgetics, antimicrobial therapy targeting secondary bacterial infection, and nutritional support. Antimicrobial therapy consisted of amoxicillin (8.75 mg/kg body weight, s.c., once daily) and metronidazole (10 mg/kg body weight, i.v., every 12 h). The patient was discharged after seven days of hospitalization in a stable clinical condition.

## 3 Diagnostic assessment

### 3.1 CPV antigen rapid testing

The dog was tested on the day of admission (November 17, 2025) using the Antigen Rapid CPV/CCV/Giardia Ag (Bionote, Hwaseong-si, Gyeonggi-do, Korea) with a weakly positive result for CPV/Ag (Supplementary Figure 2).

### 3.2 Hematological and biochemical blood examination

Peripheral blood collected from the cephalic vein (November 17, 2025) was examined by hematological method (ProCyte Dx; IDEXX Laboratories, Westbrook, ME, USA) and analysis of selected biochemical parameters (Cobas c 111; Roche Diagnostics, Basel, Switzerland). Hematological examination revealed marked leukopenia with severe neutropenia (Supplementary Table 1). Serum biochemistry demonstrated decreased total protein concentration (39.5 g/L; RI: 47–74), hyponatremia (132 mmol/L; RI: 143–151), and hypochloremia (101.2 mmol/L; RI: 110–130) (Supplementary Table 2).

### 3.3 Bacteriological and mycological cultivation

Aerobic culture of rectal swab (Central Veterinary Laboratory, Unilabs Slovakia, Likavka–Ružomberok, Slovakia) revealed a normal intestinal microbiota. Mycological culture and selective cultivation for *Campylobacter* spp. were negative.

### 3.4 Molecular detection of CPV-2 DNA and sequencing

*VP2* gene sequencing and analysis were performed to characterize the CPV-2 variant infecting the vaccinated dog. Viral DNA extracted from the rectal swab (DNeasy Blood and Tissue kit; Qiagen, Germany) was used for PCR amplification of the full-length *VP2* gene (Supplementary Method 2, Supplementary Figure 3) (13, 25). PCR products were purified (NucleoSpin Gel and PCR Clean-up Kit, Macherey-Nagel, Germany) and subjected to Sanger sequencing (Microsynth, Switzerland). The resulting *VP2* sequence was assembled (Geneious Prime v2025.2; Biomatters Ltd., New Zealand) and deposited in the GenBank database under the accession number PZ111913. BLASTn analysis against sequences available in the NCBI database confirmed CPV identity, showing 99.94%−100% pairwise nucleotide identity (query coverage 100%, *E*-value = 0.0) (Supplementary Table 3). Amino acid analysis of *VP2* identified the isolate as CPV-2c based on the presence of glutamic acid (Glu, *E*) at residue 426 (3). Comparative analysis of amino acid positions commonly used for CPV-2 characterization (25, 27–31) revealed a substitution pattern (5G, 267Y, 324I, and 370R) corresponding to the so-called “Asian” CPV-2c genetic variants (6–12) (Supplementary Table 4).

### 3.5 Phylogenetic analysis

**Figure 1:**
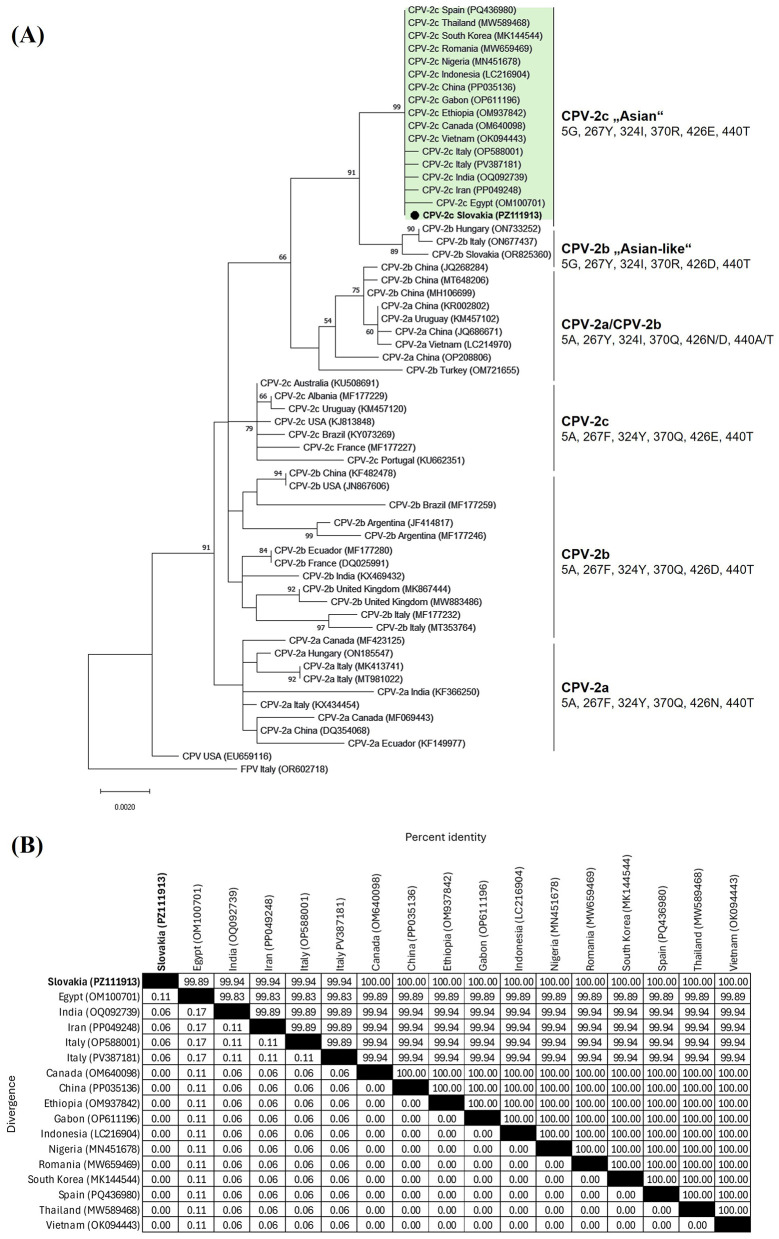
Phylogenetic and homology analysis of the CPV-2c_Slovakia isolate from this study. **(A)** The phylogenetic tree was constructed based on the complete *VP2* gene nucleotide sequences (1,755 bp) of canine parvovirus type 2 (CPV-2) obtained in this study and reference sequences retrieved from GenBank. Feline panleukopenia virus (OR602718) was included as an out-group. Bootstrap values ≥50% are depicted at their respective branches. The antigenic variants and selected amino acid residues in positions 5, 267, 324, 370, 426, and 440 are indicated. The isolate from this study (PZ111913) is highlighted. **(B)** Pairwise sequence identity between the isolate from this study and selected reference “Asian” CPV-2c strains demonstrated high genetic similarity among “Asian” CPV-2c sequences.

Phylogenetic analysis was performed based on the full-length *VP2* gene sequences (Supplementary Table 5) using the maximum likelihood (ML) method under the Tamura-three parameter model (T92) with a discrete Gamma distribution with five categories (+G) and a proportion of invariant sites (+I). The ML tree was constructed using the MEGA11 software (32). The robustness of the phylogenetic analysis was assessed by bootstrap analysis with 1,000 replicates. The phylogenetic analysis confirmed that the isolate CPV-2c_Slovakia clustered within a group corresponding to “Asian” CPV-2c strains (highlighted in Figure 1A), characterized by *VP2* amino acid residues 5G, 267Y, 324I, 370R, and 440T, with a high level of nucleotide identity with reference strains (99.83%−100%) (Figure 1B).

### 3.6 Virus isolation in cell culture

**Figure 2:**
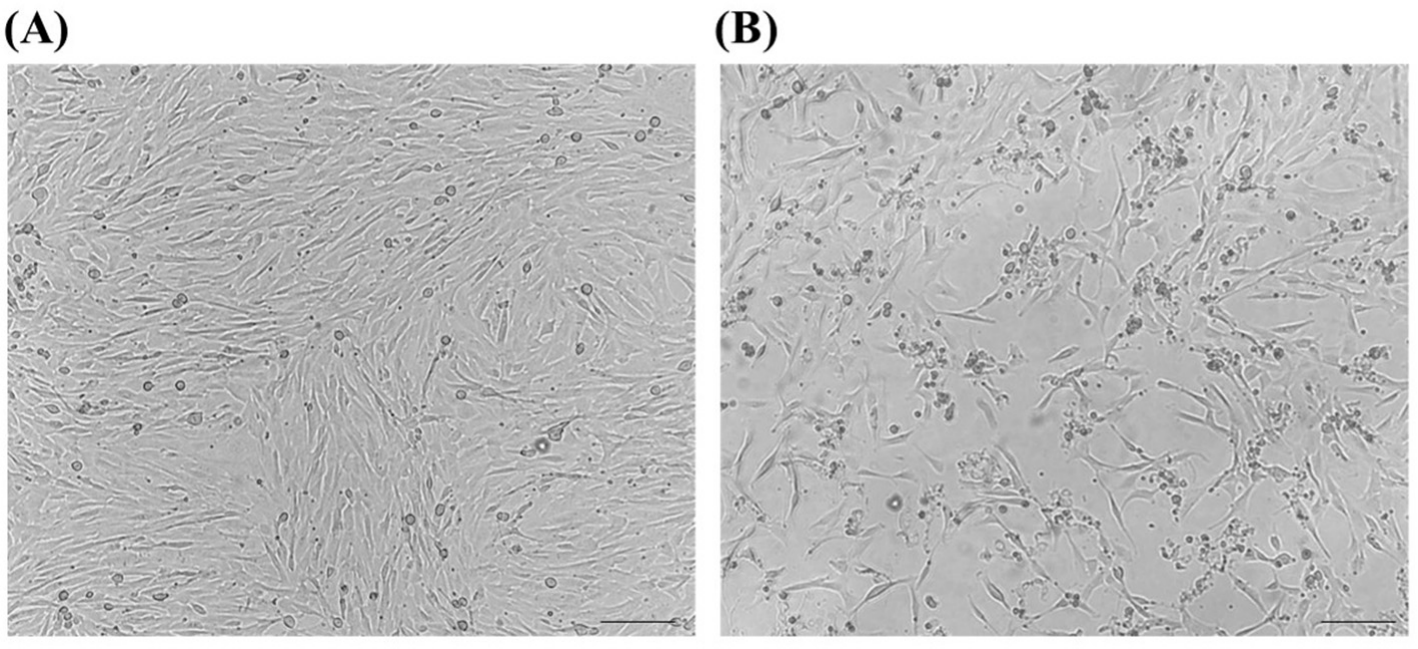
Cytopathic effect of CPV-2c_Slovakia isolate from this study on A-72 cell culture. **(A)** Uninfected A-72 cells were used as a negative control. **(B)** Cytopathic effect of CPV-2c isolate characterized by cell rounding and disruption of the monolayer. Scale bar−100 μm.

Virus isolation was performed from a rectal swab sample using A-72 (CRL-1542; ATCC, Manassas, Virginia, USA) cell culture, as previously described (33). Cell culture was monitored daily for up to 5 days for CPV-indicative cytopathic effects (CPE)_,_ characterized by cell rounding and detachment of cells from the monolayer (Figure 2). Infected cells were harvested with three freeze–thaw cycles. Viral replication in A-72 cell culture was confirmed by PCR amplification of the *VP2* gene from virus-containing culture supernatant (Supplementary Figure 4). Subsequent DNA sequencing of the cell-culture isolate confirmed the same CPV-2c genotype as detected in the original clinical sample. The titer of the isolated virus determined by 50% tissue culture infective dose (TCID_50_) assay (34) and calculated by the Reed and Muench method (35) was 10^4, 67^ TCID_50_/mL. The viral supernatant was subsequently used to estimate CPV-specific antibody titers by hemagglutination inhibition (HI) test and serum neutralization test (SNT).

### 3.7 Serological testing

#### 3.7.1 IgM and IgG antibody detection

To evaluate the humoral immune response of the patient, a blood sample was collected (November 19, 2025), and serum was used for serological testing of CPV-specific IgM and IgG antibodies (Central Veterinary Laboratory, Unilabs Slovakia, Likavka—Ružomberok, Slovakia). A high IgM antibody titer (1:2,700) was detected, whereas CPV-specific IgG antibodies were not identified (< 1:100, according to the laboratory's reference criteria) (Table 1). The same serum sample was subsequently used for HI test and SNT, as described below.

**Table 1:** Serological results of the dog from this study.

Detectend antibodies	Test	Results	Reference threshold	Interpretation	Reference
IgM	ELISA	1:2,700	>1:300	indicative of acute infection	laboratory criteria^*^
IgG	ELISA	< 1:100	>1:300	negative (not detected)	laboratory criteria^*^
HI antibodies	HI	1:640	≥1:80	protective titer	(38)
neutralizing antibodies	SNT	1:1,280	≥1:160	protective titer	(20,39)

CPV-specific IgM and IgG antibodies were determined by ELISA, hemagglutination inhibition (HI) antibodies were measured by HI assay and neutralizing antibodies by serum neutralization test (SNT). Reference thresholds indicate antibody levels considered positive or protective according to laboratory criteria and previously published studies.

^*^Central veterinary laboratory (Unilabs Slovakia; Likavka - Ružomberok, Slovakia).

#### 3.7.2 Hemagglutination inhibition assay

The viral supernatant was standardized to 8 HAU/25 μL by hemagglutination assay (HA) (36) and used as the antigen source (HA titer 1:256). HI antibodies against CPV-2 were determined using a standard HI assay (36, 37). Twofold dilutions of the serum sample (collected on November 19, 2025) in PBS (pH 7.2) (starting from 1:10) were tested. The HI titer was expressed as the reciprocal of the highest serum dilution that completely inhibited the HA activity. The detected HI titer of 1:640 is above to the threshold considered indicative of protective antibody levels (≥1:80) (38) (Table 1, Supplementary Figure 5).

#### 3.7.3 Serum neutralization test

Neutralizing antibodies against affecting CPV-2c viral variant were determined using SNT (39). Serial twofold dilutions (starting from 1:10) in MEM + 2% FBS of heat-inactivated (56 °C, 30 min) serum (collected on November 19, 2025) were mixed with 50 μL of viral suspensions containing 100 TCID_50_ of the virus. Each serum dilution was evaluated in duplicate. After 1 h of incubation at room temperature, 2 × 10^4^ A-72 cells were added to each well. The plate was incubated at 37 °C in a humidified CO_2_ atmosphere for 5 days. The determined neutralizing antibody titer of 1:1,280, expressed as the highest serum dilution that prevents the development of CPV-induced CPE, indicates antibody levels considered indicative of protection (Table 1) (20, 39).

## 4 . Discussion

In this report, we describe a clinical case of a vaccinated young dog presenting with acute hemorrhagic gastroenteritis caused by an “Asian” CPV-2c variant.

The clinical presentation at admission, characterized by vomiting, hemorrhagic diarrhea, and lethargy, was consistent with typical manifestations of parvoviral enteritis. Leukopenia is a common hematological hallmark of CPV-2 infection due to the virus-mediated destruction of bone marrow precursors, damage to the gastrointestinal tract, and depletion of lymphoid tissues (2, 40). The observed decreased leucocyte count (2.24 × 10^3^/μL) is comparable to the reported median total leukocyte count (2.35 × 10^3^/μL) described in dogs with parvovirus infection (41). The clinical findings were further supported by nonspecific biochemical abnormalities, including hypoproteinemia and electrolyte disturbances (hypochloremia and hyponatremia), which are commonly associated with gastrointestinal losses and decreased nutrient intake in dogs affected by parvoviral enteritis. Among noninvasive markers, C-reactive protein (CRP) is considered a sensitive inflammatory indicator in dogs with CPV-2 infection (42). Increased CRP levels have been associated with disease severity, survival probability, and longer hospitalization times (43). Accordingly, the elevated CRP concentration observed in the present case (77.9 mg/L) may reflect the inflammatory response associated with acute parvoviral enteritis and could be consistent with the relatively prolonged hospitalization (7 days). Although secondary bacterial translocation may occur in dogs with parvoviral enteritis due to intestinal mucosal damage, bacteriological examination did not reveal primary bacterial infection, including *Campylobacter* spp., further supporting a viral etiology of the gastrointestinal disease in this case.

The diagnosis of parvoviral enteritis in the present case was established using multiple approaches. Initial suspicion was supported by a rapid chromatographic immunological test with a weakly positive result for CPV antigen from a rectal swab sample. Previous study have demonstrated that fecal antigen testing may have limited sensitivity, particularly in cases involving CPV-2c variants (44). Moreover, the sensitivity of antigen-based assays is strongly influenced by viral load, and vaccinated dogs may shed lower amounts of virus, potentially resulting in false-negative or weakly positive results. Therefore, equivocal antigen test results should be interpreted with caution and confirmed using more sensitive molecular methods. Analysis of the deduced VP2 protein identified the isolate as a CPV-2c variant based on the presence of glutamic acid (Glu, *E*) at residue 426 (3). Furthermore, the CPV-2c_Slovakia isolate displayed the amino acid substitutions A5G, F267Y, Y324I, and Q370R, which are characteristic of CPV-2c recognized as “Asian” CPV-2c (6–8). The close relationship (>99.94%) of the CPV-2c_Slovakia isolate to previously described strains from this lineage was also supported by the phylogenetic analysis of the *VP2* gene. The detection of this lineage in a clinical case from Slovakia is epidemiologically relevant. Previous European studies have documented “Asian” CPV-2c in dogs from Italy (9, 10) and Romania (11), as well as in wildlife from Italy (45), Spain (14), and Slovakia (13), suggesting progressive dissemination of these variants across Europe. Regional circulation of CPV-2 variants carrying an “Asian” substitution pattern (A5G, F267Y, Y324I, and Q370R) is further supported by the previous detection of an “Asian-like” CPV-2b (426D) from Slovakia (25) and other European countries (30, 31, 46). Taken together, these findings indicate the co-circulation of “Asian” lineage-associated CPV-2 variants in the region and support the hypothesis that “Asian” CPV-2c is becoming established within European CPV-2 variants, extending previous observations in the region from wildlife to clinically affected vaccinated domestic dogs. Although the biological relevance of the “Asian” substitutions remains to be clarified, the ongoing expansion of these variants may indicate successful viral spread and possible fitness advantages in virus-host interactions or immune evasion, raising concerns regarding the potential impact of these emerging “Asian” CPV-2 variants on the health of the canine population. Finally, the diagnosis of CPV infection was strengthened by demonstrating the presence of infectious virus, as evidenced by the development of characteristic cytopathic effects in cell culture.

Vaccination remains the most effective measure to prevent the clinical CPV-2 infection and has markedly reduced the incidence and severity of parvoviral enteritis in dogs (47). In the present case, the dog had been vaccinated against CPV-2 and other common infectious diseases (canine distemper, adenovirosis, parainfluenza, and leptospirosis). Primary vaccination (7 weeks of age) and revaccination (11 weeks of age) were carried out within the prescribed period (4 weeks) as recommended by the manufacturer (Eurican^®^). The interval between the second and third revaccination was 4 weeks, so the third revaccination was performed before 16 weeks of age than advised (Eurican^®^). Attenuated live CPV vaccines are generally considered effective and are reported to provide cross-protection against circulating variants (17–20). However, CPV-2c was reported in association with severe disease in dogs vaccinated with a CPV-2-based vaccine (11, 22), and some studies raised concerns regarding the efficacy of vaccines in providing full protection against the “Asian” CPV-2c (6, 48). In this case, the reason(s) behind the development of clinical CPV-2 infection despite the vaccination could be explained by a combination of factors, such as (i) first of all, incompletely performed vaccine protocol and (ii) possible interference with maternally derived antibodies (MDA) (24), (iii) individual variability in immune response, or (iv) infection with hypothesized more virulent “Asian” CPV-2c which could cause more severe infections than the other types (6).

Serological tests are useful components in establishing immune status and infection dynamics. Complementary ELISA revealed a high IgM antibody titer (1:2,700) in the absence of detectable IgG antibodies. This serological profile is consistent with an acute infection-associated humoral response rather than the secondary immune response expected in an immunized animal. The lack of IgG indicates that post-vaccination protective humoral immunity had not been established or was below the detection limit at the time of testing. Possible explanations include (i) a primary immunization failure to develop detectable antibodies, potentially associated with host-related factors (delayed immune maturation, immunosuppression, individual variability in vaccine responsiveness, or MDA interference) (24), (ii) secondary immunization failure associated with waning or loss of previously acquired humoral protection (24), (iii) incomplete or improperly timed vaccination (24), or (iv) limitations of the assay used. CPV-2 seronegativity attributed, at least in part, to secondary immunization failure has also been reported in vaccinated dogs (49), suggesting that vaccination history alone may not always accurately reflect the antibody status of an individual dog at the time of clinical evaluation. However, as the serological assessment was based on a single time-point sample, seroconversion and temporal changes in IgM and IgG antibody titers could not be evaluated. Therefore, the IgM-positive/IgG-negative profile should be interpreted cautiously and in combination with the clinical course and molecular confirmation of CPV-2 infection. Additionally, HI and SNT are considered “gold standards” for specific CPV-2 serum antibody measurement (36, 39). In the serum sample, HI test and SNT performed with the homologous CPV-2c isolate revealed antibody titers of 1:640 and 1:1,280, respectively. Notably, CPV-2c has been reported to produce lower homologous neutralizing titers than heterologous ones (39). Although HI titers ≥1:80 are commonly associated with protective immunity, such titers cannot distinguish between post-vaccination immunity and an antibody response developing during active CPV-2 infection (20, 37, 39). Generally, the onset of clinical signs does not necessarily correspond to the onset of infection, and the incubation period or timing of seroconversion may vary in CPV-infected dogs (50). Since HI and SNT assess functional serum antibody activity, the high HI/neutralizing antibody titers may, at least in part, reflect the strong IgM response. Considering the high IgM and absent IgG pattern, these protective antibody titers are more consistent with a humoral response to ongoing acute CPV-2 infection rather than vaccine-induced immunity. Overall, the serological findings indicate that, despite the absence of detectable IgG antibodies, a functional antibody response was developing during infection. The clinical course, therefore, likely reflected a complex interplay between the host immune status, the infection-associated humoral response, and the infecting viral variant.

Despite confirmed CPV-2 infection, the disease did not progress to a fatal outcome, and the dog recovered following supportive treatment. The detection of an “Asian” CPV-2c lineage in this case highlights the ongoing genetic diversification of circulating CPV-2 strains and its potential implications for host–virus interactions in vaccinated dogs. Larger surveillance studies are needed to determine the frequency, distribution, and clinical relevance of these variants in the dog population.

## 5 Conclusion

This case highlights that CPV-2 infection should remain an important differential diagnosis in dogs presenting with compatible clinical signs, regardless of vaccination status. The combined use of molecular, virological, and serological methods proved essential for accurate diagnosis, particularly in cases with inconclusive antigen test results. The detection of an “Asian” CPV-2c lineage in Slovakia (Central Europe) further supports the ongoing expansion of this variant in Europe and highlights the importance of continuous molecular surveillance and monitoring of circulating CPV-2 strains even in vaccinated individuals.

## Data Availability

The datasets presented in this study can be found in online repositories. The names of the repository/repositories and accession number(s) can be found below: https://www.ncbi.nlm.nih.gov/genbank/, PZ111913.
