# Supplementary material for: Acute hemorrhagic gastroenteritis caused by an “Asian” CPV-2c variant in a vaccinated dog from Slovakia (Central Europe): a case report
Source: Front Vet Sci. 2026 Jun 24;13:1864309. doi: 10.3389/fvets.2026.1864309 (PMC13341448; doi:10.3389/fvets.2026.1864309)
Supplement: Supplementary file 1 [file Data_Sheet_1.pdf]

Supplementary Material

1 Supplementary Methods

Supplementary Method 1. Clinical examination of the patient

Clinical examination was performed according to the clinical examination protocol (1).
Dog behavior and reactions: The dog was apathetic but responsive to external stimuli.
Triage values: At admission (November 17, 2025), rectal temperature (RT) was 38.7ºC (reference value, RV: <39.2ºC), heart rate was 96 beats/min. (RV: 80-160 beats/min.), with strong and regular femoral pulses, and respiratory rate was 28 breaths/min. (RV: 15–30 breaths/min).
The dog remain afebrile throughout the entire course of hospitalization, with rectal temperatures recorded as follows: 37.0ºC (November 18, 2025:), 38.5°C (November 19, 2025), 38.5°C (November 20, 2025), 38.4°C (November 21, 2025), 38.5°C (November 22, 2025), 38.5°C (November 23, 2025).
Body Condition Scoring Systems (BCS) (2)**:** At admission was 2.5/5, indicating a suboptimal body condition, with easily palpable ribs, visible spinous processes along the spine, and a sunken lumbar area. Body weight at the time of hospitalization (November 17, 2025) was 13.1 kg. During hospitalization, the BCS deteriorated to 2/5. Body weight decreased to 11.5 kg on day 3 of hospitalization (November 19, 2025) and was 11.8 kg at discharge on day 7 (November 23, 2025).
Muscle score (MCS): MCS was 2/4 consistent with moderate muscle mass in the head, thoracic (lateral and dorsal), lumbar, and pelvic limb musculature throughout hospitalization.
Mucous membranes, capillary refill time (CRT), and hydration status: At admission, the mucous membranes were pink and moist, with a prolonged CRT (>2 s). Hydration status was moderate, with skin tenting returning to normal position within approximately 5 seconds. On November 19, 2025, the mucous membranes, including the oral mucosa, became hyperemic (brick red).
Skin and coat: The hair coat was clean and free of ectoparasites, with no evidence of pruritus. The skin appeared normal, without inflammatory or pathological changes.
Head: The head and facial region were symmetrical, without swelling or other pathological changes. No head tilt was observed.
Eyes (right and left): Both eyes were symmetrical in size and position. The eyelids were free of swelling. The conjunctivae were initially pink and later became hyperemic. Mild seromucinous discharge was observed bilaterally. The cornea was clear, and the anterior chamber, iris, and lens were bilaterally without pathological findings.
External auditory canals: The external auditory canals were pink and clean bilaterally, without excessive cerumen or abnormal discharge.
Nostrils: The nostrils were symmetrical, with no evidence of nasal discharge or swelling.
Oral cavity: At admission, the oral mucosa was pink and moist; later during hospitalization, the mucosa appeared pale. Permanent dentition was present without visible dental calculus. The hard and soft palate, tongue, and pharyngeal region were without pathological findings.
Neck: The trachea was non-irritating on palpation, and no cough was elicited. The thyroid gland was not enlarged.
Cardiovascular system: The thorax was symmetrical. Heart rate was 96 beats/min with a regular rhythm. Femoral pulses were strong, regular, and synchronous bilaterally. Cardiac auscultation revealed normal heart sounds without audible murmurs.

Respiratory tract: The thorax was symmetrical, with costo-abdominal breathing. Respiratory rate at admission was 28 breaths/min. Lung auscultation revealed normal vesicular breath sounds bilaterally, without adventitious sounds.
Abdomen: Abdominal palpation elicited mild diffuse discomfort, predominantly in the cranial and mid-abdominal regions. Increased intestinal fluid content was suspected on ballotage. Melena was present.
Urogenital apparatus: The dog was an intact male. The scrotum was free of inflammatory skin changes. The prepuce and glans penis showed no signs of inflammation or discharge. Both testes were symmetrical and fully descended into the scrotum. Urination was spontaneous, and urine appeared macroscopically yellow.
Central and peripheral nervous system: The dog was apathetic but conscious. Posture was normal, and gait was characterized by generalized weakness attributed to systemic illness. Cranial nerve examination revealed no abnormalities. Spinal reflexes in both thoracic and pelvic limbs were normal. Superficial and deep pain sensation were preserved, and postural reactions were within normal limits.
Musculoskeletal system: No swelling, deformities, or crepitus were detected on palpation of the spine or limbs. Mild generalized weakness and slight loss of muscle mass were observed during hospitalization, consistent with systemic illness.
Superficial lymph nodes: The submandibular and popliteal lymph nodes were palpable, symmetrical, not enlarged, firm, freely movable, and non-painful. Prescapular lymph nodes were not palpable.
Assessment of the presence of neoplasms and hernias: No neoplasms or hernias were detected.

Supplementary Method 2. *VP2* gene amplification

The full-length *VP2* gene (1755 bp) was amplified in four individual overlapping PCR reactions using specific primer pairs as described in our previous publication (3) (details on primers are presented in **Supplementary Table 6**). Each reaction was carried out in a total volume of 25 µL containing 12.5 µL TaKaRa Taq HS Perfect Mix (TaKaRa Bio Europe S.A.S., France), 0.2 µL of each primer (20 µM), 10.1 µL of PCR-grade water, and 2 µL of DNA template. Cycling conditions were optimized uniformly for all four reactions as follows: 35 cycles of denaturation at 94 °C for 5 s, annealing at 50 °C for 1 s, and extension at 68 °C for 15 s. As positive control, CPV-positive DNA from a previously characterized clinical sample was used (3). Amplicons (554 bp, 541 bp, 563 bp, and 539 bp) were stained with GelRed (Biotinum, USA), separated by electrophoresis on 1.0% agarose gel in 1 × borax buffer, and visualized with a UVP transilluminator M-15V (Analytik Jena, Germany) (**Supplementary Figure 3**).

2 Supplementary Figures and Tables

2.1 Supplementary Figures

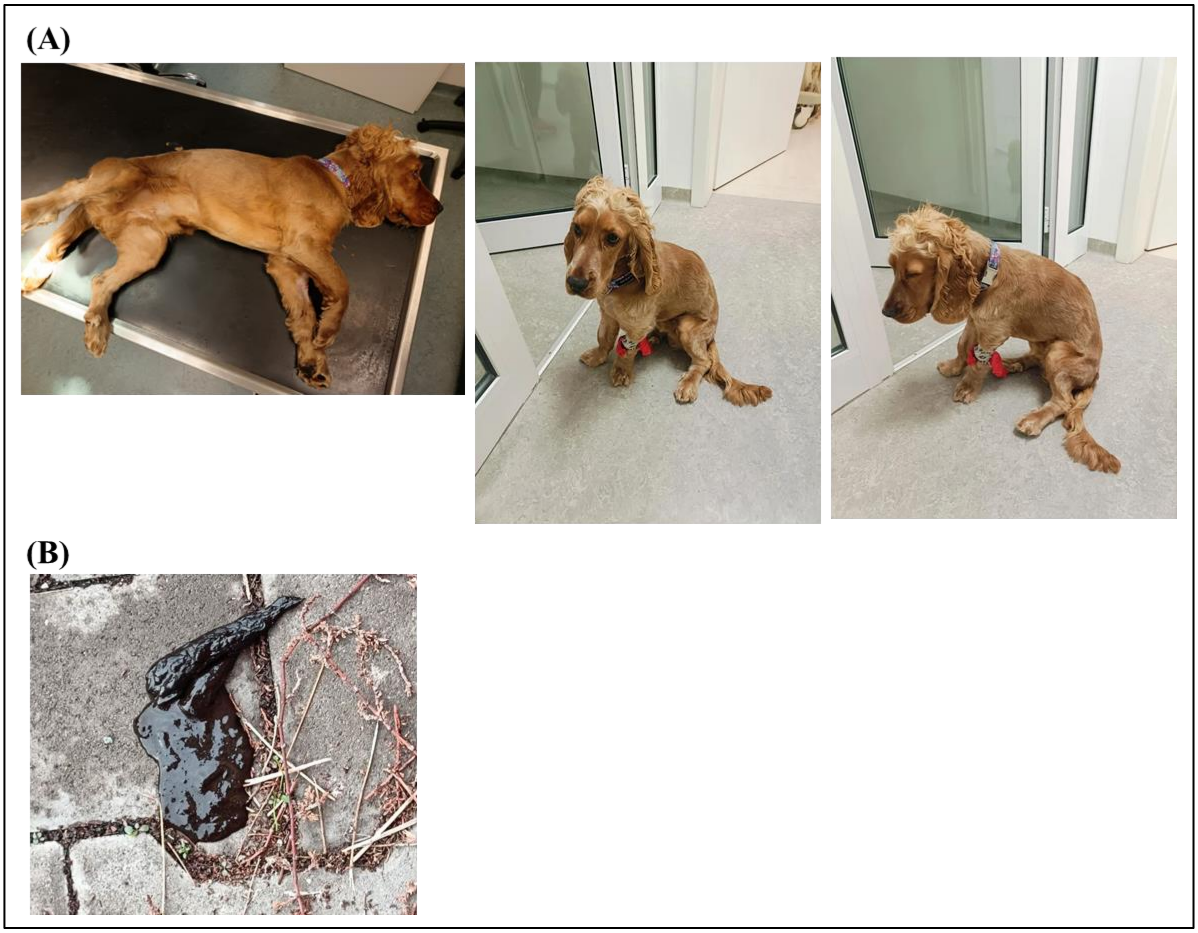

Supplementary Figure 1. An 8-month-old male English Cocker Spaniel with parvovirus disease (November 17, 2025). **(A)** The patient showed lethargy and reduced activity. **(B)** Hemorrhagic diarrhea.

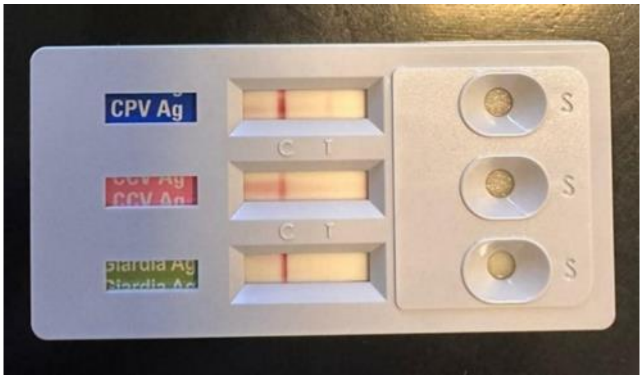

Supplementary Figure 2. A weakly positive rapid antigen test for CPV antigen from a rectal swab sample (November 17, 2025).

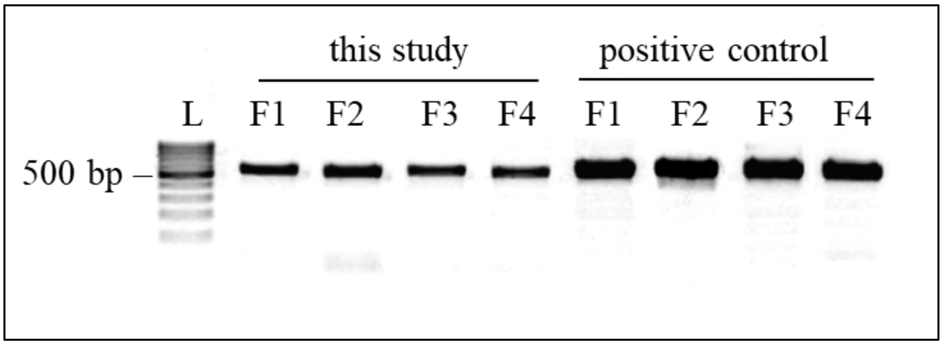

Supplementary Figure 3. The amplification of the full-length *VP2* gene (1,755 bp) from the clinical sample. Agarose gel electrophoresis of overlapping PCR fragments covering the full-length *VP2* gene (1,755 bp) amplified from viral DNA extracted from the rectal swab sample. Lanes: L – DNA ladder (GeneRuler 100 bp); F1 – F4 overlapping *VP2* gene fragments used for sequence assembly (554 bp, 541 bp, 563 bp, 539 bp); positive control – CPV+ sample (3).

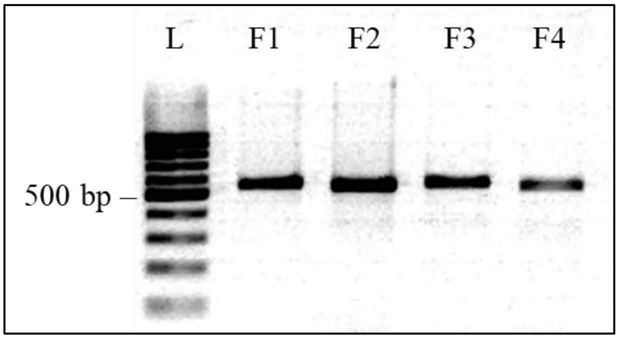

Supplementary Figure 4. Molecular confirmation of CPV-2 replication in A-72 cells. PCR amplification of *VP2* gene from cell culture supernatant confirming viral replication in cell culture. Lanes: L – DNA ladder (GeneRuler 100 bp); F1: frame 1 (554 bp); F2: frame 2 (541 bp); F3: frame 3 (563 bp); F4: frame 4 (539 bp).

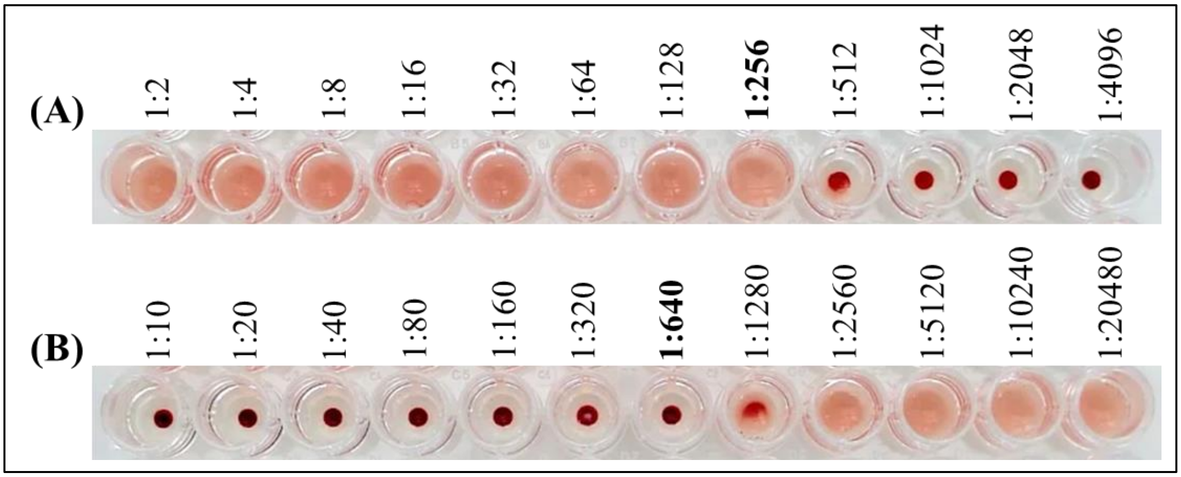

Supplementary Figure 5. H Hemagglutination (HA) and hemagglutination-inhibition (HI) assays of serum from a vaccinated dog with confirmed CPV-2 infection. **(A)** HA assay performed with the virus-containing culture supernatant from this study showing an HA titer of 1:256 (in bold). **(B)** HI assay performed with serial twofold dilutions of the tested serum (starting at 1:10) with complete inhibition of hemagglutination observed up to a serum dilution of 1:640 (in bold), corresponding to the HI antibody titer.

Supplementary Table 1. Results of blood count and differential blood count.

Blood count	Conclusion	Result	Unit	Reference interval
RBC	OK	6.13	10^{12}/L	5.65-8.87
HCT	OK	41.4	%	37.3-61.7
HGB	OK	15.1	g/dL	13.1-20.5
MCV	OK	67.5	fL	61.6-73.5
MCH	OK	24.6	Pg	21.2-25.9
MCHC	OK	36.5	g/dL	32.0-37.9
RDW	OK	14.8	%	13.6-21.7
%RETIC	OK	0.2	%	180.0-500.0
RETIC	OK	13.5	K/µL	10.0-110.0
RETIC-HGB	OK	23.0	Pg	22.3-29.6
**WBC**	**-**	**2.24**	**10^9/L**	**5.05-16.76**
% NEU		3.6	%	
% LYM		56.3	%	
% MONO		32.1	%	
% EOS		8.0	%	
% BASO		0.0	%	
**NEU**	**-**	**0.08**	**10^9/L**	**2.95-11.64**
LYM	OK	1.26	10^9/L	1.05-5.10
MONO	OK	0.72	10^9/L	0.16-1,12
EOS	OK	0.18	10^9/L	0.06-1,23
BASO	OK	0.00	10^9/L	0.00-0.10
PLT	OK	169	K/µL	148-484
MPV	OK	13.0	fL	8.7-13.2
PDW	OK	13.8	fL	9.1-19.4
PCT	OK	0.22	%	0.14-0.46

Values outside the reference intervals are highlighted in bold. Hematological examination revealed leukopenia with severe neutropenia. Abbreviations: RBC – Red Blood Cells, HCT – Hematocrit, HGB – Hemoglobin, MCV – Mean Corpuscular Volume, MCH – Mean Corpuscular Hemoglobin, MCHC – Mean Corpuscular Hemoglobin Concentration, RDW – Red Blood Cell Distribution Width, %RETIC – Percentage of Reticulocytes, RETIC – Reticulocytes, RETIC-HGB – Reticulocyte Hemoglobin Ratio, WBC – White Blood Cells, NEU – Neutrophils, LYM – Lymphocytes, MONO – Monocytes, EOS – Eosinophils, BASO – Basophils, PLT – Platelets, MPV – Mean Platelet Volume, PDW – Platelet Distribution Width, PCT – Plateletcrit.

Supplementary Table 2. Results of the biochemistry test.

Parameter	Conclusion	Result	Unit	Reference interval
Glucose	OK	5.34	mmol/L	3.6-5.8
**Urea**	**-**	**3.87**	**mmol/L**	**3.97-8.05**
**Creatinine**	**-**	**38.1**	**µmol/L**	**46-88**
Phosphorus	OK	1.23	mmol/L	0.90-1.91
Calcium	OK	2.08	mmol/L	2.05-2.86
**Total protein**	**-**	**39.5**	**g/L**	**47-74**
ALT	OK	0.49	µkat/l	<0.949
ALP	OK	1.19	µkat/l	<1.24
AST	OK	0.22	µkat/l	<0.6
Pancreatic amylase	OK	6.91	µkat/l	<7.21
Lipase	OK	0.5	µkat/l	<1.66
Potassium	OK	3.82	mmol/l	3.5-5.1
**Sodium**	**-**	**132**	**mmol/l**	**143-151**
**Chlorides**	**-**	**101.2**	**mmol/L**	**110-130**

Values outside the reference intervals are highlighted in bold. Biochemistry examination revealed decreased total protein concentration, hyponatremia, hypochloremia, and decreased values of urea and creatinine. Abbreviations: ALT – Alanine Aminotransferase, ALP – Alkaline Phosphatase, AST – Aspartate Aminotransferase.

Supplementary Table 3. List of sequences producing significant alignments with the full-length *VP2* gene (1,755 bp) sequence obtained in this study using the NCBI BLASTn algorithm (accessed February 24, 2025).

GenBank accession number	Pairwise identity [%]	Query coverage [%]	E value	CPV-2 variant	Origin	Year of collection
ON322787.1	100.00	100	0.0	CPV-2c	China	2020
PP035136.1	100.00	100	0.0	CPV-2c	China	2023
MH711894.1	100.00	100	0.0	CPV-2c	Thailand	2016
LC216904.1	100.00	100	0.0	CPV-2c	Indonesia	2013
PV296092.1	100.00	100	0.0	CPV-2c	China	2023
ON322777.1	100.00	100	0.0	CPV-2c	China	2020
MT648203.1	100.00	100	0.0	CPV-2c	China	2019
ON322786.1	100.00	100	0.0	CPV-2c	China	2020
ON322798.1	100.00	100	0.0	CPV-2c	China	2020
OQ092740.1	100.00	100	0.0	CPV-2c	India	2022
ON322831.1	100.00	100	0.0	CPV-2c	China	2020
ON322842.1	100.00	100	0.0	CPV-2c	China	2021
ON322826.1	100.00	100	0.0	CPV-2c	China	2020
ON322816.1	100.00	100	0.0	CPV-2c	China	2020
PP035139.1	100.00	100	0.0	CPV-2c	China	2023
ON322814.1	100.00	100	0.0	CPV-2c	China	2020
ON322759.1	100.00	100	0.0	CPV-2c	China	2020
PP035147.1	100.00	100	0.0	CPV-2c	China	2021
OK094440.1	100.00	100	0.0	CPV-2c	Vietnam	2019
PP035126.1	100.00	100	0.0	CPV-2c	China	2022
ON322810.1	100.00	100	0.0	CPV-2c	China	2020
MN451678.1	100.00	100	0.0	CPV-2c	Nigeria	2018
PV296094.1	100.00	100	0.0	CPV-2c	China	2021
ON322753.1	100.00	100	0.0	CPV-2c	China	2020
MN451682.1	100.00	100	0.0	CPV-2c	Nigeria	2018
ON322811.1	100.00	100	0.0	CPV-2c	China	2020
ON322812.1	100.00	100	0.0	CPV-2c	China	2020
PP035138.1	100.00	100	0.0	CPV-2c	China	2023
MT840294.1	100.00	100	0.0	CPV-2c	Nigeria	2018
MT840293.1	100.00	100	0.0	CPV-2c	Nigeria	2018
MZ614964.1	100.00	100	0.0	CPV-2c	China	2019
ON322806.1	100.00	100	0.0	CPV-2c	China	2020
PP035112.1	100.00	100	0.0	CPV-2c	China	2021
OM640098.1	100.00	100	0.0	CPV-2c	Canada	2018
OP588002.1	100.00	100	0.0	CPV-2c	Italy	2021
OK094443.1	100.00	100	0.0	CPV-2c	Vietnam	2020
ON322802.1	100.00	100	0.0	CPV-2c	China	2020
MW589468.1	100.00	100	0.0	CPV-2c	Thailand	2020

MW650830.1	100.00	100	0.0	CPV-2c	China	2020
MK144544.1	100.00	100	0.0	CPV-2c	South Korea	2017
MH476587.1	100.00	100	0.0	CPV-2c	China	2017
ON322847.1	100.00	100	0.0	CPV-2c	China	2020
PQ436980.1	100.00	100	0.0	CPV-2c	Spain	2024
PP035145.1	100.00	100	0.0	CPV-2c	China	2021
MT106228.1	100.00	100	0.0	CPV-2c	Vietnam	2017
MF510157.1	100.00	100	0.0	CPV-2c	Italy	2017
ON322778.1	100.00	100	0.0	CPV-2c	China	2020
MH476583.1	100.00	100	0.0	CPV-2c	China	2017
MW811189.1	100.00	100	0.0	CPV-2c	China	2020
MH711902.1	100.00	100	0.0	CPV-2c	Thailand	2016
ON322836.1	100.00	100	0.0	CPV-2c	China	2020
MG013488.1	100.00	100	0.0	CPV-2c	China	2017
MZ506743.1	100.00	100	0.0	CPV-2c	China	2019
OM523076.1	100.00	100	0.0	CPV-2c	China	2021
ON322789.1	100.00	100	0.0	CPV-2c	China	2020
ON322807.1	100.00	100	0.0	CPV-2c	China	2020
OP611196.1	100.00	100	0.0	CPV-2c	Gabon	2019
ON322785.1	100.00	100	0.0	CPV-2c	China	2020
PP035132.1	100.00	100	0.0	CPV-2c	China	2023
ON322761.1	100.00	100	0.0	CPV-2c	China	2020
OK094439.1	100.00	100	0.0	CPV-2c	Vietnam	2018
OK384308.1	100.00	100	0.0	CPV-2c	China	2019
ON322837.1	100.00	100	0.0	CPV-2c	China	2021
ON322760.1	100.00	100	0.0	CPV-2c	China	2020
PP035128.1	100.00	100	0.0	CPV-2c	China	2023
ON322839.1	100.00	100	0.0	CPV-2c	China	2021
PQ442333.1	100.00	100	0.0	CPV-2c	China	2019
OP588001.1	99.94	100	0.0	CPV-2c	Italy	2021
ON322821.1	99.94	100	0.0	CPV-2c	China	2020
PP049248.1	99.94	100	0.0	CPV-2c	Iran	2021
PX146866.1	99.94	100	0.0	CPV-2c	Slovakia	2023
PQ442341.1	99.94	100	0.0	CPV-2c	China	2019
PQ442350.1	99.94	100	0.0	CPV-2c	China	2019
OM523071.1	99.94	100	0.0	CPV-2c	China	2021
OR724817.1	99.94	100	0.0	CPV-2c	China	2022
OR724780.1	99.94	100	0.0	CPV-2c	China	2021
OQ092739.1	99.94	100	0.0	CPV-2c	India	2022
PV296090.1	99.94	100	0.0	CPV-2c	China	2023
PP035123.1	99.94	100	0.0	CPV-2c	China	2022
ON322754.1	99.94	100	0.0	CPV-2c	China	2020
OR724797.1	99.94	100	0.0	CPV-2c	China	2021
MW815496.1	99.94	100	0.0	CPV-2c	China	2020
OR724783.1	99.94	100	0.0	CPV-2c	China	2021
OQ092734.1	99.94	100	0.0	CPV-2c	India	2022

MW728967.1	99.94	100	0.0	CPV-2c	China	2020
OM523075.1	99.94	100	0.0	CPV-2c	China	2021
PX392195.1	99.94	100	0.0	CPV-2c	China	2020
ON322758.1	99.94	100	0.0	CPV-2c	China	2020
PQ178754.1	99.94	100	0.0	CPV-2c	China	2022
PV387181.1	99.94	100	0.0	CPV-2c	Italy	2022
OM057679.1	99.94	100	0.0	CPV-2c	China	2021
PP035148.1	99.94	100	0.0	CPV-2c	China	2021
OR724765.1	99.94	100	0.0	CPV-2c	China	2020
ON322797.1	99.94	100	0.0	CPV-2c	China	2020
ON322757.1	99.94	100	0.0	CPV-2c	China	2020
ON322788.1	99.94	100	0.0	CPV-2c	China	2020
PP035124.1	99.94	100	0.0	CPV-2c	China	2022
MT840292.1	99.94	100	0.0	CPV-2c	Nigeria	2018
PX392217.1	99.94	100	0.0	CPV-2c	China	2020
OR724769.1	99.94	100	0.0	CPV-2c	China	2020

Supplementary Table 4. Amino acid profile observed in VP2 sequence of the CPV-2c_Slovakia isolate identified in this study and reference CPV-2 strains.

CPV-2 variant	Country	Year	Accession number	Amino acid position of VP2 gene					
				5	267	324	370	426	440
**CPV-2**	USA	1979	EU659116	A	F	Y	Q	N	T
**CPV-2a**	Italy	2018	MT981022	A	F	Y	Q	N	T
	Slovakia	2023	PX146838	A	F	Y	Q	N	T
	Hungary	2020	ON185547	A	F	Y	Q	N	T
	China	2004	DQ354068	A	Y	Y	Q	N	T
	China	2015	MH106699	A	Y	I	Q	N	T
	China	2010	KF6384000	A	Y	I	Q	N	A
	Vietnam	2013	LC214970	A	Y	I	Q	N	A
**CPV-2b**	Portugal	2013	KR559895	A	F	Y	Q	D	T
	Italy	2019	MT353761	A	F	Y	Q	D	T
	Slovakia	2025	PX146850	A	F	Y	Q	D	T
	China	2009	KF482478	A	Y	I	Q	D	T
	China	2018	MT648206	A	Y	I	Q	D	A
	Turkey	2019	OM721655	A	Y	I	Q	D	A
**CPV-2b "Asian-like"**	Italy	2022	ON677437	G	Y	I	R	D	T
	Hungary	2021	ON733252	G	Y	I	R	D	T
	Slovakia	2022	OR825360	G	Y	I	R	D	T
**CPV-2c**	Australia	2015	KU508691	A	F	Y	Q	E	T
	Italy	2019	MT353760	A	F	Y	Q	E	T
	Brazil	2015	KY073269	A	F	Y	Q	E	T
**CPV-2c "Asian"**	China	2023	PP035136	G	Y	I	R	E	T
	Italy	2022	PV387181	G	Y	I	R	E	T
	Romania	2019	MW659469	G	Y	I	R	E	T
	**Slovakia**	**2025**	**PZ111913**	**G**	**Y**	**I**	**R**	**E**	**T**

The sequence analyzed in this study is displayed in bold. Abbreviations: A–Ala–Alanine, D–Asp–Aspartic acid, E–Glu–Glutamic acid, F–Phe–Phenylalanine, G–Gly–Glycine, I–Ile–Isoleucine, N–Asn–Asparagine, Q–Gln–Glutamine, R–Arg–Arginine, S–Ser–Serine, T–Thr–Threonine, Y–Tyr–Tyrosine.

Supplementary Table 5. Reference strains used in this study for phylogenetic analysis (accessed February 25, 2025).

GenBank accession number	Variant	Country
EU659116	CPV-2	USA
MF423125	CPV-2a	Canada
MF069443	CPV-2a	Canada
KF14997	CPV-2a	Ecuador
ON185547	CPV-2a	Hungary
DQ354068	CPV-2a	China
JQ686671	CPV-2a	China
KR002802	CPV-2a	China
OP208806	CPV-2a	China
KF366250	CPV-2a	India
KX434454	CPV-2a	Italy
MK413741	CPV-2a	Italy
MT981022	CPV-2a	Italy
KM457102	CPV-2a	Uruguay
LC214970	CPV-2a	Vietnam
JF414817	CPV-2b	Argentina
MF177246	CPV-2b	Argentina
MF177259	CPV-2b	Brazil
KX774252	CPV-2b	Brazil
MF177280	CPV-2b	Ecuador
DQ025991	CPV-2b	France
ON733252	CPV-2b	Hungary
KF482478	CPV-2b	China
JQ268284	CPV-2b	China
MH106699	CPV-2b	China
MT648206	CPV-2b	China
KX469432	CPV-2b	India
MF177232	CPV-2b	Italy
MT353761	CPV-2b	Italy
ON677437	CPV-2b	Italy
OR825360	CPV-2b	Slovakia
OM721655	CPV-2b	Turkey
MK867444	CPV-2b	United Kingdom
MW883486	CPV-2b	United Kingdom
JN867606	CPV-2b	USA
MF177229	CPV-2c	Albania
KU508691	CPV-2c	Australia
KY073269	CPV-2c	Brazil
OM640098	CPV-2c	Canada
OM100701	CPV-2c	Egypt
OM937842	CPV-2c	Ethiopia
MF177227	CPV-2c	France
OP611196	CPV-2c	Gabon
PP035136	CPV-2c	China

OQ092739	CPV-2c	India
LC216904	CPV-2c	Indonesia
PP049248	CPV-2c	Iran
OP588001	CPV-2c	Italy
PV387181	CPV-2c	Italy
MN451678	CPV-2c	Nigeria
KU662351	CPV-2c	Portugal
MW659469	CPV-2c	Romania
MK144544	CPV-2c	South Korea
PQ436980	CPV-2c	Spain
MW589468	CPV-2c	Thailand
KM457120	CPV-2c	Uruguay
KJ813848	CPV-2c	USA
OK094443	CPV-2c	Vietnam
OR602718	FPV	Italy

Abbreviations: CPV – canine parvovirus; FPV – feline panleukopenia virus (used as an outgroup in phylogenetic analysis)

Supplementary Table 6. Primers used for the amplification of full-length *VP2* gene of canine parvovirus in a clinical sample.

Primer	Sequence (5´ - 3´)	Position*	Amplicon size	Reference
CPV-F<br>CPV-VP2-INT-R1	AGAGACAATCTTGCACCAAT<br>CTATCTAATGCAACCATCAATG	2768–2787<br>3300-3321	554 bp	(3)
CPV-VP2-INT-F1<br>CPV-VP2-INT-R2	GTTGCATTTAGTTAGTTTTGAACA<br>ACCACGTCTTTTATCTTGTTG	3190–3213<br>3710–3730	541 bp	
CPV-VP2-INT-F2<br>CPV-VP2-INT-R3	GATTGTAAACCATGTAGACTAACA<br>GCAGTTAAAGGACCATAAGTA	3590–3613<br>4132–4152	563 bp	
CPV-VP2-INF-F3<br>CPV-R	GAAGATATCCAGAAGGAGATTGG<br>ATGTTAATATAATTTTCTAGGTGCT	4005–4027<br>4519–4543	539 bp	

*Primer positions are referred to the reference sequence of the canine parvovirus 2 complete genome (accession no.: NC_001539).

References

1. Taylor SM. *Small Animal Clinical Techniques - E-Book: Small Animal Clinical Techniques - E-Book*. Elsevier Health Sciences. (2020). 322 p.

2. Laflamme D. Development and Validation of a Body Condition Score System for Dogs. *Canine Pract* (1997) 22:10–15.

3. Pelegrinová A, Petroušková P, Korytár Ľ, Ondrejková A, Drážovská M, Vojtek B, Mojžišová J, Prokeš M, Kostičák M, Zákutná Ľ, et al. The first evidence of Asian-like CPV-2b in Slovakia in a vaccinated dog with an acute fatal course of parvovirus infection: a case report. *Vet Res Commun* (2024) 48:3253–3262. doi: 10.1007/s11259-024-10492-z
