# Supplementary material for: Acute hemorrhagic gastroenteritis caused by an “Asian” CPV-2c variant in a vaccinated dog from Slovakia (Central Europe): a case report
Source: Front Vet Sci. 2026 Jun 24;13:1864309. doi: 10.3389/fvets.2026.1864309 (PMC13341448; doi:10.3389/fvets.2026.1864309)
Supplement: Supplementary file 2 [file Supplementary_file_1.pdf]

CARE case report guidelines

CARE Checklist of information to include when writing a case report

(cc) BY-NC-ND

Topic	Item	Checklist item description	Reported on Line
Title	1	The diagnosis or intervention of primary focus followed by the words "case report"	Title
Key Words	2	2 to 5 key words that identify diagnoses or interventions in this case report, including "case report"	Keywords
Abstract (no references)	3a	Introduction: What is unique about this case and what does it add to the scientific literature?	Abstract
	3b	Main symptoms and/or important clinical findings	Abstract
	3c	The main diagnoses, therapeutic interventions, and outcomes	Abstract
	3d	Conclusion—What is the main "take-away" lesson(s) from this case?	Abstract
Introduction	4	One or two paragraphs summarizing why this case is unique (**may include references**)	Introduction
Patient Information	5a	De-identified patient specific information.	Case description
	5b	Primary concerns and symptoms of the patient.	Case description
	5c	Medical, family, and psycho-social history including relevant genetic information	Case description
	5d	Relevant past interventions with outcomes	Case description
Clinical Findings	6	Describe significant physical examination (PE) and important clinical findings.	Case description
Timeline	7	Historical and current information from this episode of care organized as a timeline	Supplementary File
Diagnostic Assessment	8a	Diagnostic testing (such as PE, laboratory testing, imaging, surveys).	Diagnostic assessment
	8b	Diagnostic challenges (such as access to testing, financial, or cultural)	Diagnostic assessment
	8c	Diagnosis (including other diagnoses considered)	Diagnostic assessment
	8d	Prognosis (such as staging in oncology) where applicable	Case description
Therapeutic Intervention	9a	Types of therapeutic intervention (such as pharmacologic, surgical, preventive, self-care)	Case description
	9b	Administration of therapeutic intervention (such as dosage, strength, duration)	Case description
	9c	Changes in therapeutic intervention (with rationale)	-
Follow-up and Outcomes	10a	Clinician and patient-assessed outcomes (if available)	Case description
	10b	Important follow-up diagnostic and other test results	Diagnostic assessment
	10c	Intervention adherence and tolerability (How was this assessed?)	Case description
	10d	Adverse and unanticipated events	-
Discussion	11a	A scientific discussion of the strengths AND limitations associated with this case report	Discussion
	11b	Discussion of the relevant medical literature **with references**.	Discussion
	11c	The scientific rationale for any conclusions (including assessment of possible causes)	Discussion
	11d	The primary "take-away" lessons of this case report (without references) in a one paragraph conclusion	Discussion
Patient Perspective	12	The patient should share their perspective in one to two paragraphs on the treatment(s) they received	-
Informed Consent	13	Did the patient give informed consent? Please provide if requested	Yes ☒ No ☐
